# Unlocking Apoptotic Pathways: Overcoming Tumor Resistance in CAR‐T‐Cell Therapy

**DOI:** 10.1002/cam4.70283

**Published:** 2024-10-08

**Authors:** Zhanna Zhang, Manqi Su, Panruo Jiang, Xiaoxia Wang, Xiangmin Tong, Gongqiang Wu

**Affiliations:** ^1^ Department of Hematology Dongyang Hospital Affiliated to WenZhou Medical University Jinhua Zhejiang China; ^2^ Department of Central Laboratory School of Medicine, Affiliated Hangzhou First People's Hospital, WestLake University Zhejiang Hangzhou China

**Keywords:** apoptotic pathways, CAR‐T‐cell therapy resistance, tumor, tumor microenvironment

## Abstract

**Background:**

Chimeric antigen receptor (CAR)‐T‐cell therapy has transformed cancer treatment, leading to remarkable clinical outcomes. However, resistance continues to be a major obstacle, significantly limiting its efficacy in numerous patients.

**Objectives:**

This review critically examines the challenges associated with CAR‐T‐cell therapy, with a particular focus on the role of apoptotic pathways in overcoming resistance.

**Methods:**

We explore various strategies to sensitize tumor cells to CAR‐T‐cell‐mediated apoptosis, including the use of combination therapies with BH3 mimetics, Mcl‐1 inhibitors, IAP inhibitors, and HDAC inhibitors. These agents inhibit anti‐apoptotic proteins and activate intrinsic mitochondrial pathways, enhancing the susceptibility of tumor cells to apoptosis. Moreover, targeting the extrinsic pathway can increase the expression of death receptors on tumor cells, further promoting their apoptosis. The review also discusses the development of novel CAR constructs that enhance anti‐apoptotic protein expression, such as Bcl‐2, which may counteract CAR‐T cell exhaustion and improve antitumor efficacy. We assess the impact of the tumor microenvironment (TME) on CAR‐T cell function and propose dual‐targeting CAR‐T cells to simultaneously address both myeloid‐derived suppressor cells (MDSCs) and tumor cells. Furthermore, we explore the potential of combining agents like PPAR inhibitors to activate the cGAS‐STING pathway, thereby improving CAR‐T cell infiltration into the tumor.

**Conclusions:**

This review highlights that enhancing tumor cell sensitivity to apoptosis and increasing CAR‐T cell cytotoxicity through apoptotic pathways could significantly improve therapeutic outcomes. Targeting apoptotic proteins, particularly those involved in the intrinsic mitochondrial pathway, constitutes a novel approach to overcoming resistance. The insights presented herein lay a robust foundation for future research and clinical applications aimed at optimizing CAR‐T cell therapies.

## Graphical abstract

CAR‐T‐cell therapy has shown remarkable promise in cancer treatment but faces significant resistance challenges. This review examines strategies to overcome resistance by targeting apoptotic pathways, including novel combination therapies and CAR constructs. Additionally, it highlights the impact of the tumor microenvironment and the potential of combining strategies to improve CAR‐T cell function and infiltration into tumors.

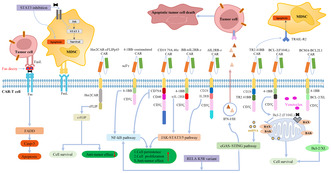

## 1 Introduction

**FIGURE 1:**
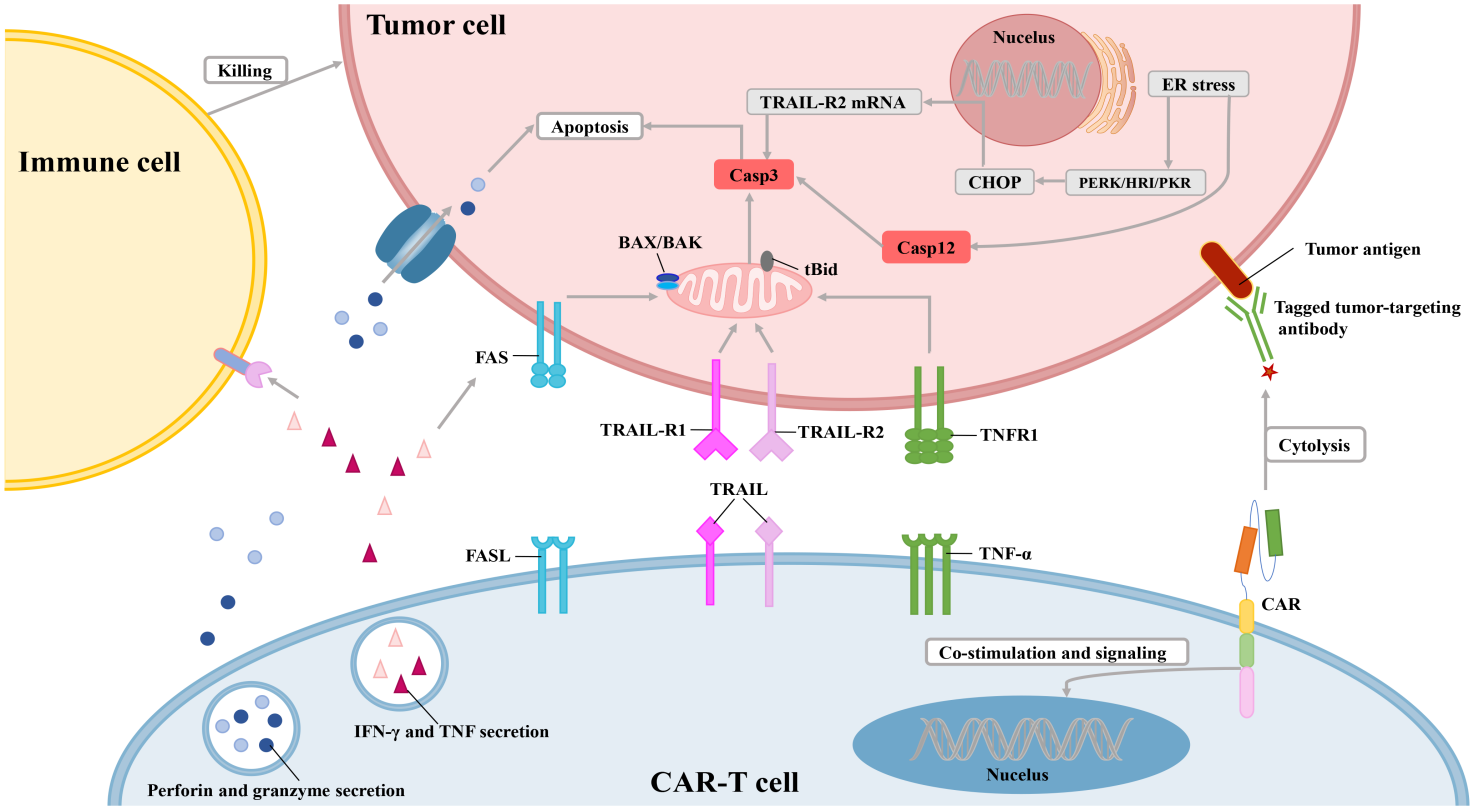
The apoptotic pathways involved in CAR‐T‐cell‐mediated tumor cell death, including the intrinsic mitochondrial pathway, extrinsic death receptor pathway, common pathway, and intrinsic endoplasmic reticulum pathway. Upon recognizing a tumor‐associated antigen, CAR‐T cells establish an immune synapse with the target cell, leading to the activation of immune responses and subsequent induction of tumor cell apoptosis.

Chimeric antigen receptor (CAR)‐T‐cell therapy has yielded remarkably effective and durable clinical responses in recent years [1]. Through genetic engineering, T cells can express CARs on their surface and transform into CAR‐T cells. CARs are engineered chimeric molecules comprising three parts: a single‐chain fragment variable (scFv), a transmembrane domain, and CD3ζ. The extracellular scFv selectively recognizes and targets tumor antigens. The intracellular CD3ζ domain, serving as the primary activating domain, initiates signaling pathways in T cells. Additional costimulatory domains, such as CD28 or 4‐1BB, are typically incorporated into CAR constructs to further enhance T‐cell activation [2]. CAR allows T cells to specifically target antigens without MHC restriction. As a result, CAR‐T cells are vigorously activated and potently destroy tumor cells [3]. CAR‐T‐cell therapy has demonstrated remarkable efficacy against hematologic malignancies and solid tumors. Unfortunately, many patients who receive CAR‐T‐cell therapy could experience resistance. Solid tumors exhibit greater heterogeneity in antigen expression. Thus, they are more prone to resistance. The following challenges to the mechanisms of CAR‐T‐cell therapy resistance have been described thus far: (1) defective CAR‐T cell functional structure [4, 5], (2) tumor cell evasion of CAR‐T‐cell monitoring through antigen escape [6, 7], (3) immunosuppressive tumor microenvironments (TMEs) [8], (4) on‐target and off‐tumor toxicity [9, 10], (5) inefficient transportation and permeation of CAR‐T cells [11, 12], (6) heterogeneity of tumor‐associated antigens [13, 14], (7) upregulation of death ligands and inhibitory receptors [15, 16, 17], and (8) T‐cell depletion and RNA epigenetic changes [18]. Recent data suggest that intrinsic resistance mechanisms weaken CAR‐T‐cell anticancer efficiency. To elucidate the novel mechanisms of cancer insensitivity to CAR‐T‐cell therapy, understanding the cytotoxicity of CAR‐T cells is crucial. When a CAR‐T cell identifies a tumor‐associated antigen, it establishes an immune synapse with the target cell (Figure 1). This interaction activates the CAR‐T cell, leading to the release of cytotoxic substances, including perforin and granzymes. Granzymes enter tumor cells through channels created by perforin, initiating intrinsic apoptosis by damaging mitochondria and activating caspases. Furthermore, CAR‐T cells enhance the expression of ligands that interact with death receptors on tumor cells, activating the extrinsic apoptotic pathways and promoting caspase‐driven tumor cell death. Moreover, CAR‐T cells release IFN‐γ and TNF, which further sensitize tumor cells to apoptosis. This is achieved by reducing the activation threshold for both FasL/TRAIL‐mediated and mitochondrial apoptosis pathways. As a result, cancer cells are more readily induced to undergo programmed cell death. IFN‐γ also activates and enhances the effector functions of immune cells, including cytotoxic T lymphocytes (CTLs) and NK cells, making them more effective at recognizing and destroying tumor cells [19]. Additionally, complex mechanisms of apoptosis are involved in programmed cell death [20]. The intrinsic mitochondrial pathway, the extrinsic death receptor pathway, and the intrinsic endoplasmic reticulum pathway are key components of apoptotic pathways. Recent studies have shown that apoptotic pathways, especially the death receptor axis, are critical to CAR‐T‐cell‐mediated killing [21, 22].

In this review, we first elucidate the apoptotic pathways involved. In summary, recent studies have documented that apoptotic pathways and CAR‐T‐cell resistance are closely related. Finally, we discuss potential strategies to alleviate resistance by improving CAR‐T‐cell immunotherapy.

## 2 Apoptosis

To prolong the survival time and mitigate resistance, it is essential to further our comprehension of the mechanisms driving CAR‐T‐cell cytotoxicity. Apoptosis is a critical homeostasis mechanism in multicellular organisms that can eliminate unwanted or abnormal cells, play a key role in embryonic development, maintain homeostasis, and respond to normal physiological processes such as pathogens. In the process of apoptosis, caspases are crucial [23, 24]. The mitochondrial and death receptor pathways eventually converge into a common pathway to execute apoptosis (Figure 1).

### 2.1 The Intrinsic Mitochondrial Pathway

The intrinsic mitochondrial pathway is triggered by multiple internal stresses, such as low oxygen levels, severe oxidative damage, and irreparable genetic damage. Mitochondrial permeability can be enhanced by these incentives, which enable the transfer of pro‐apoptotic proteins to the cytoplasm. The nomenclature “B Cell Lymphoma 2 (Bcl‐2) family proteins” stems from the discovery of the Bcl‐2 gene, initially discovered in individuals afflicted with follicular non‐Hodgkin lymphoma (NHL) [25]. The Bcl‐2 family of proteins is divided into three distinct categories. The first group consists of BH3‐only proteins, which are crucial for initiating apoptosis. The second group includes anti‐apoptotic proteins that prevent cell death and promote survival. Lastly, pro‐apoptotic effector proteins play a direct role in executing the cell death program. These groups are essential in controlling mitochondrial outer membrane permeabilization (MOMP) [24]. On the mitochondrial surface, BH3‐only proteins like Bim, Bad, Puma, and Noxa play a crucial role in triggering apoptosis by engaging with pro‐apoptotic proteins like BAX and BAK. These BH3‐only proteins contain a specific domain, known as the BH3 domain, which facilitates interactions within the Bcl‐2 family members. This domain allows them to either directly activate pro‐apoptotic proteins or inhibit anti‐apoptotic proteins, such as Bcl‐XL, Mcl‐1, Bcl‐W, Bcl2A1, and Bcl‐2. Bim and Puma inhibit all listed anti‐apoptotic proteins, while Noxa blocks only Bcl2A1 and Mcl‐1, and Bad inhibits Bcl‐2, Bcl‐XL, and Bcl‐W [26]. Subsequently, BAK and BAX form oligomers, leading to MOMP, triggering intrinsic apoptosis [23]. During MOMP, proteins like cytochrome c (cyt c) and Smac located in the mitochondrial intermembrane space are released into cytosol. Smac can efficiently and rapidly activate caspases by inhibiting caspase inhibitory proteins such as XIAP. Cytosolic cyt c participates in forming the apoptosome complex with procaspase‐9 and Apaf1, ultimately activating caspase‐9, which triggers caspase‐7, caspase‐6, and caspase‐3, executing intrinsic apoptosis [27, 28, 29]. Recent studies have shown that mitochondrial DNA (mtDNA) can also be transferred to the cytoplasm following MOMP. In the absence of caspases, cytoplasmic mtDNA activates the cGAS‐STING pathway. The cGAS‐STING pathway plays a crucial role in the detection of cytosolic DNA and the initiation of innate immune responses. Upon recognizing mtDNA in the cytoplasm, cGAS catalyzes the production of cGAMP, which then binds to STING. This interaction triggers downstream signaling pathways that lead to the activation of transcription factors, such as IRF3 and NF‐κB, resulting in the production of type I interferons (IFN‐I) and other pro‐inflammatory cytokines [30, 31]. Almost all types of cells can produce IFN‐I in an immune response. Once IFN‐I binds to the heterodimers of IFNAR1 and IFNAR2, it can activate the JAK/STAT1 pathway. The activation of STAT1 can further trigger the Fas/TRAIL‐mediated apoptotic pathways, as well as enhance the activation of BAX and BAK. Caspases can suppress the cGAS‐STING pathway, reducing inflammatory reactions, and result in rapid cell apoptosis to preserve more normal cells during cell death [32, 33].

### 2.2 The Extrinsic Death Receptor Pathway

The extrinsic apoptotic pathway, driven by death receptors, plays a vital role in triggering apoptosis in cancer cells. The binding of ligands to these receptors on the surface of target cells initiates the apoptotic process by activating the associated signaling pathways. Upon recognition and stimulation by CTLs, there is an upregulation of death receptors. These receptors, belonging to the TNF receptor superfamily, have death domains located on their intracellular segments. Upon ligands trimerizing their respective receptors, the apoptosis signal is transduced to intracellular signaling pathways through death domains. Numerous ligand and death receptor pairs are present on the cell surface. FasL/Fas, TRAIL/TRAIL‐R1/R2, and TNF‐α/TNFR1 are among the most well‐characterized examples [34, 35, 36].

When ligands attach to their corresponding receptors, such as Fas and TRAIL‐R, adaptor proteins like Fas‐associated death domain (FADD) are recruited. This recruitment links procaspase‐8 to FADD through a death effector domain (DED), leading to the assembly of the death‐inducing signaling complex (DISC). Within the DISC, caspase‐8 undergoes proteolytic activation. Activated caspase‐8 subsequently triggers cell death by activating downstream caspases [37]. Caspase‐8 also cleaves Bid, a pro‐apoptotic protein, converting it into tBid, which then moves to the mitochondria [38]. Upon localization in the mitochondria, tBid predominantly engages with other Bcl‐2 family proteins via its BH3 domain, thereby establishing a close connection between the extrinsic death receptor pathway and the mitochondrial pathway.

The function of TNFR1 exhibits greater complexity compared to that of Fas and TRAIL‐R, attributed to the expanded diversity of proteins constituting the TNFR1 complex. When TNF‐α links to TNFR1, recruitment of TRADD to TNFR1 occurs. The conformational change in TNFR1 enables the recruitment of RIPK1 and TRAF2, which further constitute complex I near the cytomembrane. TRAF2 recruits the ubiquitin ligases cIAP1 and cIAP2, which can produce polyubiquitin (Ub) chains and participate in docking to the LUBAC, TAK/TAB, and NEMO/IKK complexes. Activated Complex I, which contains IKK, can trigger the MAPK and NF‐κB pathways, which cause the transcription of a few genes involved in cell survival (Bcl‐2, Bcl‐XL, and cFLIP) and proinflammatory genes. The activation of NF‐κB signaling does not contribute to apoptosis [39]. TRADD, including the signalosome, can also recruit FADD, which transforms the signalosome into the complex II. Complex II is a death‐inducing signaling pathway capable of activating procaspase‐8, subsequently initiating caspase‐3 activation and apoptosis [34, 36]. Moreover, JNK is an important MAPK protein family member that can enhance apoptosis.

### 2.3 The Common Pathway

A series of activated caspases is involved in the apoptotic process. Caspase‐8 is crucial in the extrinsic apoptosis pathway, which is triggered by death receptors on the cell surface. On the other hand, caspase‐9 is central to the intrinsic apoptosis pathway, which involves signals from the mitochondria. Both of these pathways ultimately lead to the activation of caspase‐3, a key enzyme in executing cell death. Activated caspase‐3 then targets deoxyribonuclease, which impedes further caspase activation and facilitates nuclear apoptosis. Additionally, downstream caspases cleave inhibitory subunits of the endonuclease family, DNA repair proteins, protein kinases, and cytoskeletal proteins. They further impact the signaling pathways, cell division, and the cytoskeleton, all result in morphological alterations during apoptosis [40].

### 2.4 The Intrinsic Endoplasmic Reticulum Pathway

The intrinsic ER pathway represents a third mechanism for inducing apoptosis, yet its intricacies remain incompletely elucidated. The unfolded protein response (UPR) mediates ER proteostasis surveillance. Cellular stressors such as hypoglycemia, hypoxia, and free radicals can impair the ER, prompting the UPR to inhibit protein synthesis and initiate apoptosis. This process involves signaling from the ER to the cytosol and nucleus regarding protein folding issues. Consequently, procaspase‐12 dissociates from TRAF2, becomes activated, and converts into caspase‐12, which plays a critical role in the execution of apoptosis [41]. In addition, apoptosis triggered by ER stress engages pro‐apoptotic proteins from the Bcl‐2 family within the intrinsic mitochondrial pathway and also involves the apoptosome complex and caspase‐3. However, there is still controversy over the connection between the intrinsic ER pathway and the intrinsic mitochondrial pathway [42]. ER stress can interact with and activate the TRAIL pathway, along with caspase‐8, leading to the initiation of cell death [43].

## 3 Enhancing the Sensitivity of Tumor Cells

### 3.1 Strategies Targeting the Intrinsic Mitochondrial Pathway to Induce Apoptosis

**FIGURE 2:**
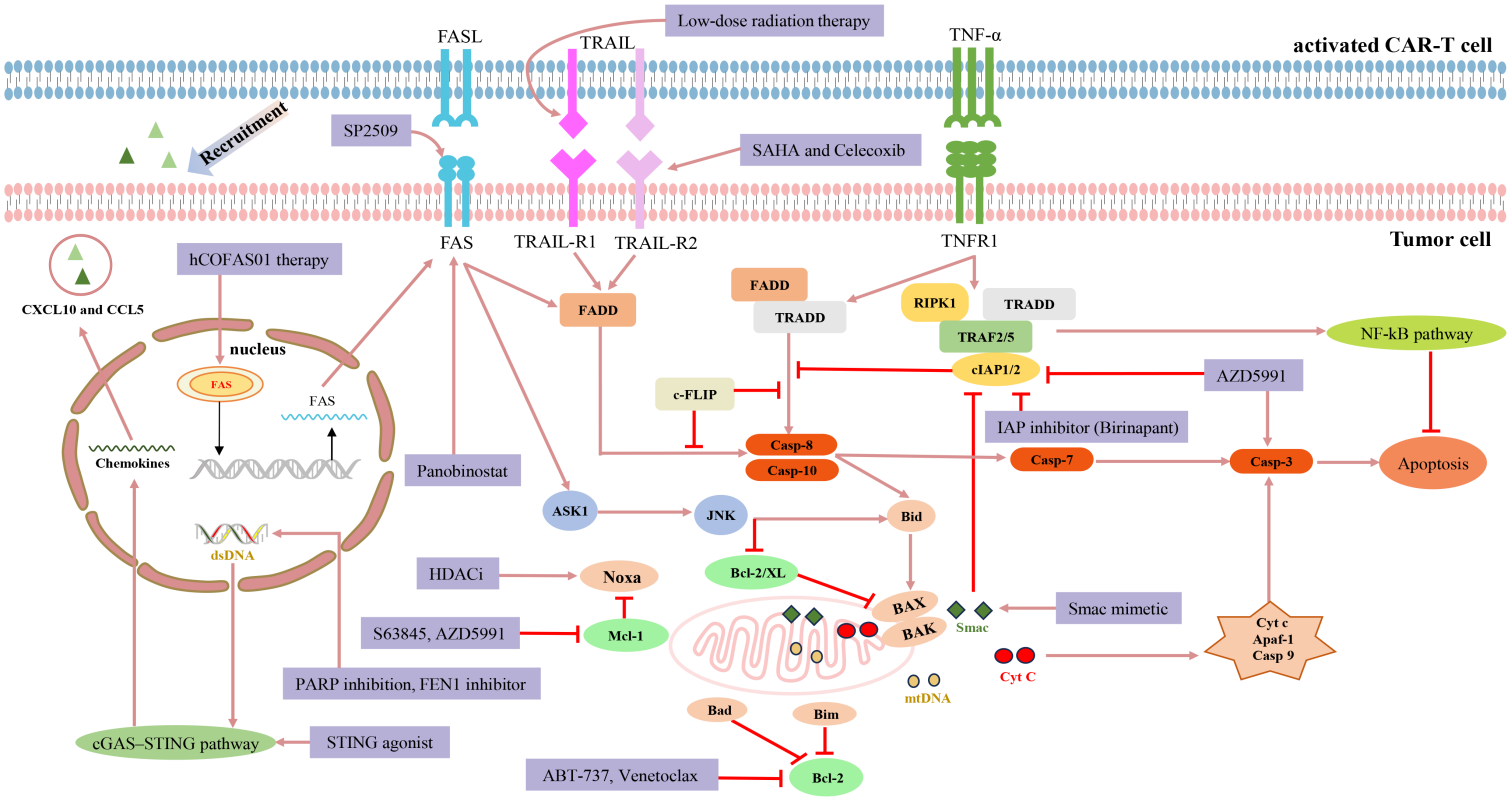
Apoptotic pathways and strategies to enhance the sensitivity of tumor cells to CAR‐T‐cell therapy. The apoptotic pathways include the intrinsic mitochondrial pathway, extrinsic death receptor pathway, and intrinsic endoplasmic reticulum pathway. Bcl‐2 inhibitors, Mcl‐1 inhibitors, KDM1A inhibitors, IAP inhibitors, HDACi, and Smac mimetics play a role in enhancing the sensitivity of tumor cells to apoptosis and augmenting CAR‐T‐cell cytotoxic functions.

Bcl‐2, Bcl‐XL, and Mcl‐1 can contribute to therapeutic resistance by inhibiting intrinsic apoptosis signaling, specifically through the blockade of BAK and BAX at the mitochondrial membrane [44, 45]. To counteract this resistance, a viable strategy is to combine CAR‐T‐cell therapy with agents that inhibit anti‐apoptotic proteins from the Bcl‐2 family or with compounds that enhance the activity of pro‐apoptotic proteins like BAX. Ranjan and colleagues conducted a high‐throughput drug screening utilizing a co‐culture system involving Nalm6 leukemic cells and CAR‐T cells to identify candidate molecules for modulating CAR‐T‐cell function. Among the compounds tested, BH3 mimetics (e.g., venetoclax and ABT‐737), IAP inhibitors (such as birinapant), Mcl‐1 inhibitors (like S63845), and Smac mimetics have been shown to make cancer cells more vulnerable to attack by CAR‐T cells [46] (Figure 2).

#### 3.1.1 BH3 Mimetics

The creation of ABT‐737 represented the initial example of a small compound meticulously engineered to target the hydrophobic pockets of BCL‐2 and BCL‐XL, the exact sites where pro‐apoptotic BH3‐only proteins bind. Administering ABT‐737 to tumor cells prior to CAR‐T‐cell therapy can reinstate the intrinsic apoptotic pathways disrupted by CAR‐T‐cell therapy in B‐cell malignancy cell lines. This approach enhances the efficacy of cell killing and helps preserve the viability of effector cells [47]. By screening 29 pro‐apoptotic drugs in the B‐cell precursor leukemia cell line NALM6, researchers identified venetoclax, a selective BCL‐2 inhibitor, as significantly enhancing CAR‐T cell efficacy by promoting apoptosis in the target cancer cells. CAR‐T cells can kill 47%–63% of target cells on their own. However, when combined with BCL‐2 inhibitors, which help overcome the anti‐apoptotic mechanisms of the tumor, their killing efficiency improves significantly, reaching 75%–88%. This increased effectiveness is due to the inhibitors enhancing the ability of CAR‐T cells to induce cell death in cancer cells. In an animal model of diffuse large B‐cell lymphoma using the OCI‐Ly18 cell line, the combined treatment of venetoclax and CAR‐T cells led to a better response of tumor cells to interferon. This combination not only significantly slowed down tumor progression but also markedly increased the survival rates of the mice [48]. In an experimental murine model of lymphoma or leukemia, CAR‐T cells engineered to overexpress the Bcl‐2 protein were administered in combination with venetoclax. Venetoclax specifically targets the Bcl‐2 protein in tumor cells, neutralizing its protective effect and thereby reactivating the apoptosis mechanism within the tumor cells [49].

#### 3.1.2 Mcl‐1 Inhibitors

Mcl‐1, a key pro‐survival protein, has garnered significant attention as a therapeutic target due to its upregulation across numerous cancer types and its pivotal role in conferring drug resistance. Mcl‐1 restrains BAK, Bcl‐2 inhibits BAX, and Bcl‐XL restrains both BAK and BAX [50]. Patients with mantle cell lymphoma who exhibit resistance to CAR‐T‐cell therapy show a notable increase in Mcl‐1 expression. This suggests that high Mcl‐1 levels may lead to the reduced treatment effectiveness, indicating a potential link between elevated Mcl‐1 and therapy resistance. The small molecule AZD5991, a macrocyclic inhibitor specifically targeting Mcl‐1, binds to an Mcl‐1 groove, displacing BAK and inducing apoptosis. AZD5991 amplifies the apoptotic response by downregulating levels of IAPs and caspase‐3 via a BAK‐dependent mechanism. When combined with CAR‐T‐cell therapy, AZD5991 significantly slows down the growth rate of tumors and extends the survival period in two aggressive models of mantle cell lymphoma derived from patient samples [51].

#### 3.1.3 IAP Inhibitors

In numerous cancers, the overproduction of inhibitors of apoptosis (IAP) proteins is a common occurrence. This excessive presence of IAPs can lead to poorer prognoses, as these proteins prolong cell survival by preventing the activation of caspases, which are crucial for initiating cell death. Among the eight identified IAP proteins in humans, XIAP, c‐IAP1, c‐IAP2, and ML‐IAP are particularly notable for their prominent role in preventing apoptosis. The second mitochondria‐derived activator of caspase (Smac) is crucial for counteracting the effects of inhibitor of IAP proteins. Smac is released from mitochondria into the cytoplasm, where it directly antagonizes IAPs, thereby alleviating their suppression of the apoptosome. Smac is especially important for binding to proteins such as XIAP, c‐IAP1, and c‐IAP2, particularly by preventing XIAP from suppressing caspases 3, 7, and 9. Although c‐IAP1 and c‐IAP2 are less effective at inhibiting caspases on their own, they can promote apoptosis by binding to Smac, which prevents Smac from interacting with XIAP and thereby facilitates the activation of caspases. IAP proteins influence cell survival through various mechanisms. To counteract their effects and enhance treatment efficacy, significant research has focused on integrating CAR‐T‐cell therapy with inhibitors of IAP proteins or SMAC mimetics [52, 53]. This approach aims to enhance the susceptibility of cancer cells to CAR‐T‐cell therapy by counteracting the protective effects of IAPs and promoting a more effective immune response.

Birinapant, an IAP inhibitor, mimics the action of SMAC by binding to and inhibiting IAPs, thereby promoting the apoptosis of cancer cells. It enhances bystander cell death triggered by CAR‐T cells in antigen‐negative glioblastoma multiforme cells and boosts apoptosis in primary glioblastoma multiforme culture models with antigen heterogeneity when treated with CAR‐T cells. Furthermore, birinapant does not impair the functionality of CAR‐T cells. On the contrary, it amplifies their effectiveness by facilitating the activation of NF‐κB signaling pathways [54]. Despite the hurdles and the requirement for additional clinical trials to establish their efficacy, the prospect of enhancing anti‐tumor immune responses by integrating IAP inhibitors and SMAC mimetics with CAR‐T‐cell therapy holds significant promise.

#### 3.1.4 Targeting Noxa to Induce Apoptosis

Noxa functions as a sensitizer protein by regulating apoptosis indirectly. While it does not directly activate BAX or BAK, it crucially modulates cell death by inhibiting anti‐apoptotic proteins such as Mcl‐1, Bcl‐XL, and BCL‐2. Moreover, Noxa can displace activators like BIM or BAX/BAK monomers, thereby setting off the apoptotic cascade. Anti‐apoptotic members possess four BH domains that form a binding groove, which sequesters pro‐apoptotic activators or sensitizers, including BH3‐only proteins, along with BAK and BAX. Noxa binds tightly to Mcl‐1 and effectively inhibits its function. Through unbiased genome‐wide CRISPR/Cas9 screening of CAR‐T‐cell‐resistant cells, investigators demonstrated that the deletion of Noxa in B‐NHL can inhibit CAR‐T‐cell‐induced endogenous apoptosis in target cells, leading to the development of resistance to CAR‐T‐cell therapy in tumor cells. Noxa expression is either absent or low in approximately 38% of NHL patients. Among those with low Noxa levels, around 70% experience disease progression within 6 months, compared to only 15% of patients with high Noxa expression who develop the disease within the same time frame. Histone deacetylase inhibitors (HDACi) are epigenetic modulators that exert their anticancer effects primarily by inducing apoptosis. These agents can also serve as valuable additions to CAR‐T‐cell therapy, thereby enhancing its therapeutic effectiveness. Specifically, HDACi can elevate the expression of Noxa, a factor that makes tumor cells more vulnerable to the destructive effects of CAR‐T cells. Consequently, targeting Noxa pharmacologically may improve the outcomes of CAR‐T‐cell therapy by overcoming resistance [55].

### 3.2 Strategies Targeting the Extrinsic Pathway to Induce Apoptosis

The extrinsic pathway of apoptosis is triggered by the activation of death receptors, which are part of the TNF receptor family. Key death receptors involved in this process include Fas, TRAIL‐R1/R2, and TNFR1. These receptors are essential for conveying apoptotic signals from the cell surface to the internal apoptotic machinery following their interaction with specific ligands. Tumor cells frequently evade CAR‐T‐cell‐mediated immune surveillance by downregulating the expression of these death receptors [56, 57] (Figure 2).

#### 3.2.1 Targeting FAS to Induce Apoptosis

Fas and FasL demonstrate significant antitumor activity in vitro and have been explored as potential therapeutic targets in vivo. Fas‐mediated apoptosis occurs independently of antigen recognition. Mamonkin et al. revealed that CAR‐T cells express more Bcl‐2, while malignant T cells express more Bid, making T‐lymphoma and T‐ALL cell lines more sensitive to Fas‐mediated apoptosis [56]. To better understand survival outcomes in patients undergoing CAR‐T‐cell therapy for malignant melanoma, researchers analyzed gene expression profiles of tumor cells. This analysis revealed that individuals with higher levels of Fas expression generally experienced extended survival compared to those with lower Fas levels [58]. However, their activation has also been associated with adverse effects, causing ischemic and hemorrhagic damage in various tissues, leading to acute liver failure and septic shock in animal models [59]. To avoid these adverse effects, researchers are exploring more precise gene‐targeting strategies and regulatory mechanisms to safely harness the antitumor potential of Fas and FasL. The tumor suppressor gene p53 promotes cell apoptosis through upregulating the expression of death receptors. Therefore, wild‐type p53‐expressing cells synthesize more p53 proteins after exposure to DNA‐damaging agents or various cytotoxic agents [59, 60]. Inhibition of KDM1A by SP2509 can modify chromatin structure and gene expression, which may result in the activation of TP53. The activation of TP53 can enhance its tumor‐suppressive functions, including Fas‐mediated DNA repair. This enhancement increases the tumor's sensitivity to CAR‐T‐cell therapy [61]. Resistance of human melanoma to apoptosis through death receptors was reversed by using cationic lipid nanoparticle encapsulation to introduce DNA into melanoma cells, thereby restoring Fas expression [58].

Diminished Fas‐mediated apoptosis in tumor cells can reduce the effectiveness of CAR‐T‐cell therapy. Effective CAR‐T‐cell treatment often relies on robust Fas‐mediated signaling within cancer cells, as it helps to address variability in target antigen presentation. Therefore, leveraging epigenetic modifications to enhance Fas‐mediated cytotoxicity offers a valuable strategy to combat antigen downregulation and enhance the outcomes of CAR‐T‐cell therapies.

#### 3.2.2 Targeting TRAIL‐R1/R2 to Induce Apoptosis

TRAIL, a transmembrane trimeric glycoprotein, serves as an extracellular ligand for TRAIL‐R1/R2. TRAIL can be cleaved by enzymes called metalloproteases, resulting in the formation of a soluble form of the protein. This soluble TRAIL is then able to bind to TRAIL receptors located in various tissues throughout the body. Expressed on the membrane of CAR‐T cells, TRAIL helps to modulate the innate immune system to target and eliminate cancer cells [57]. CD19 CAR‐T cells show decreased efficacy in targeting NHL cells in the presence of TRAIL inhibitors. When NHL cells develop resistance to CD19 CAR‐T‐cell therapy, their susceptibility to TRAIL‐induced apoptosis is significantly lower compared to that of the original cell line [62]. Moreover, CAR‐T cells have the ability to trigger apoptosis in tumor cells via the granzyme–perforin pathway. Targeting TRAIL‐R1/R2 to directly activate the extrinsic apoptotic pathway could be an effective approach to improve CAR‐T‐cell therapy. By focusing on these specific receptors, therapies can potentially boost the ability of CAR‐T cells to induce cell death in tumors, thereby enhancing the overall effectiveness of the treatment. Additionally, low‐dose radiation therapy can mitigate tumor cell escape from CAR‐T cells by increasing the expression of TRAIL‐associated genes [63]. Non‐steroidal anti‐inflammatory drugs (NSAIDs) have been identified for their capacity to induce ER stress and oxidative stress, thereby leading to apoptosis in cancer cells. For instance, indomethacin and COX2 inhibitors like celecoxib have been shown to increase the TRAIL‐R2 levels on tumor cells while also enhancing TRAIL production in CAR‐T cells through oxidative stress induction. This means that these drugs help CAR‐T cells recognize and attack cancer cells [64]. Furthermore, NSAIDs can diminish PGE2‐mediated immunosuppression within the TME, thereby fostering a more supportive setting for CAR‐T cells to function. Their use has been explored to enhance the effectiveness of immunotherapy in individuals with B‐cell malignancies [65]. Despite their potential to enhance CAR‐T‐cell therapy outcomes, NSAIDs offer a cost‐effective and accessible approach. However, TRAIL's role in inducing apoptosis in CAR‐T cells indicates that COX inhibitors may have conflicting effects when used in conjunction with CAR‐T‐cell therapy [66]. While they boost the cytotoxic impact on cancer cells, they may also impair CAR‐T cell function and quantity by inhibiting NF‐κB pathways [62]. Therefore, these medications should be administered with caution in patients undergoing CAR‐T‐cell therapy. These findings have reinvigorated interest in TRAIL‐based strategies as a promising avenue to address resistance in CAR‐T‐cell therapies.

## 4 Increasing the Cytotoxicity Of Car‐T Cells

**FIGURE 3:**
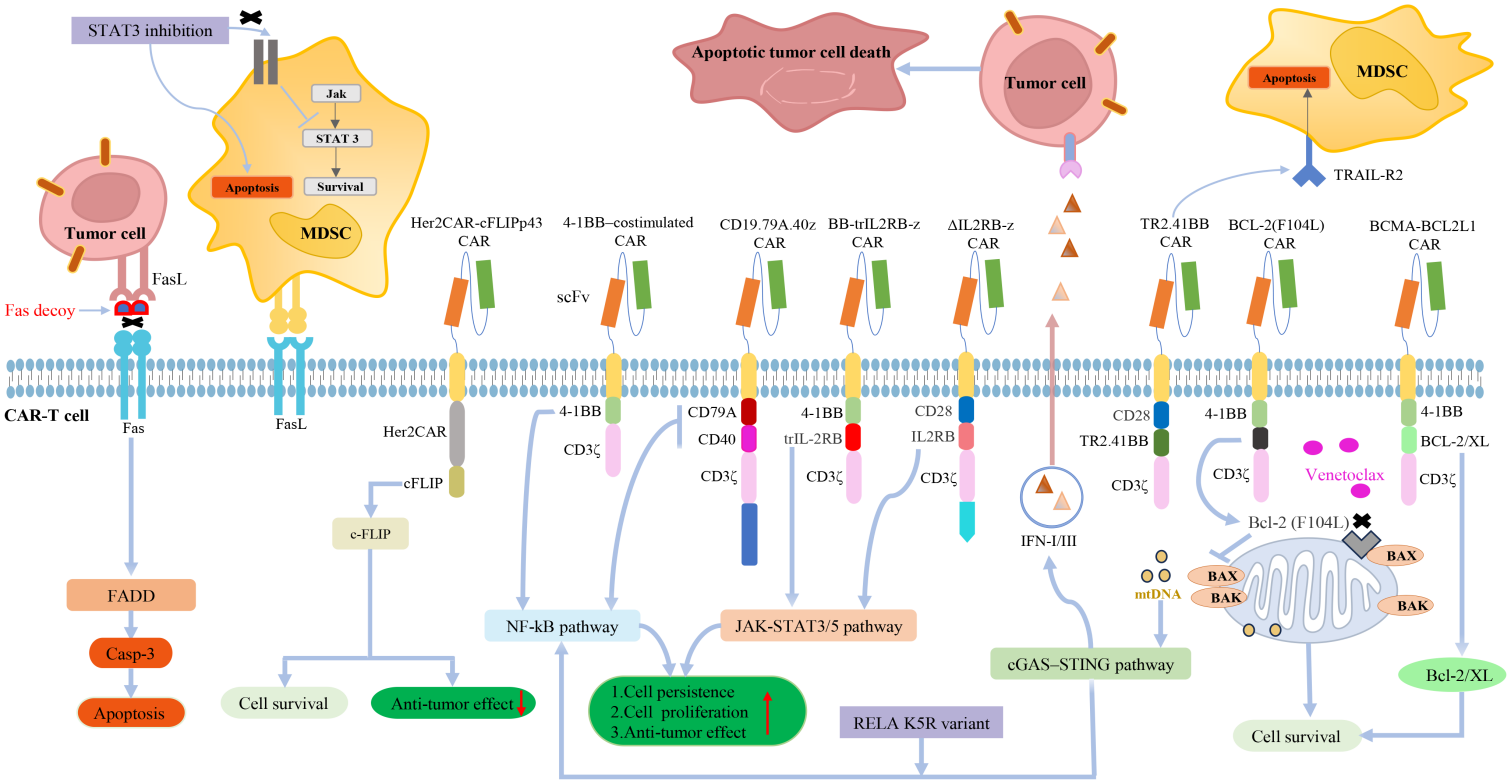
Interactions between tumor cells and CAR‐T cells. Immunosuppressive MDSCs inhibit CAR‐T‐cell activity, including apoptosis. Some novel CAR cassettes can inhibit apoptotic pathways and enhance the antitumor effect of CAR‐T cells.

Enhancing the sensitivity of tumor cells to apoptosis through both intrinsic and extrinsic pathways is crucial for improving the overall efficacy of cancer therapies. However, these strategies should be complemented by approaches that concurrently enhance the cytotoxic potential of CAR‐T cells. The subsequent section examines how targeting specific apoptosis‐regulating pathways can not only augment CAR‐T cell efficacy but also enable them to overcome tumor‐induced immunosuppression (Figure 3). By addressing these dual aspects—sensitizing tumor cells and bolstering CAR‐T cell function—a stronger and more sustained anti‐tumor response can be achieved.

### 4.1 Strategies Targeting the Intrinsic Pathway to Inhibit Apoptosis

#### 4.1.1 Overcoming the Limitation of Bcl‐2 Inhibitors on CAR‐T Cells

Notably, anti‐apoptotic proteins play a crucial role in safeguarding CAR‐T cells from apoptosis triggered by death receptors, thereby preventing activation‐induced cell death [67]. As a result, anti‐apoptotic molecules will have a positive impact on CAR‐T cells. In clinical trial NCT02030834, researchers examined gene expression levels in T cells from 38 B‐NHL patients treated with CD19 CAR‐T cells [68]. Patients who achieved remission exhibited higher transcriptional levels of Bcl‐2 in T cells compared to those who did not respond to the therapy. This indicates that higher Bcl‐2 levels could improve patient survival during CAR‐T‐cell treatment. In certain venetoclax‐sensitive lymphoma models, combining venetoclax with CAR‐T cells led to a marked increase in antitumor efficacy. However, inhibitors of anti‐apoptotic proteins often induce toxicity and reduce CAR‐T cell viability. Thus, these inhibitors could be used to presensitize malignant cells before CAR‐T‐cell treatment, making them suitable for application prior to adoptive transfer. Lee et al. developed a venetoclax‐resistant CAR‐T‐cell line by overexpressing the mutant form of Bcl‐2 (F104L) [48]. This mutation removes the effects of venetoclax on CAR‐T cells, allowing them to maintain their function despite venetoclax treatment. Moreover, Mamonkin and colleagues found that during treatment with Bcl‐2 inhibitors, CAR‐T cells exhibited a substantial upregulation in Bcl‐2 expression. This elevated expression appeared to provide sufficient protection to safeguard CAR‐T cells from undergoing apoptosis [56]. Despite this, developing appropriate strategies to promote the production of anti‐apoptotic proteins in CAR‐T cells, aiming to boost their persistence and functionality within the body. Wang and colleagues addressed this by incorporating Bcl‐2 into a second‐generation CAR structure, which not only improved CAR‐T‐cell survival but also extended the lifespan of lymphoma xenograft mouse models [69]. Utilizing CITE‐seq technology, Ranjan et al. discovered that CAR‐T‐cell therapy in resistant patients leads to reduced Bcl‐XL expression. To address this, they designed an innovative CAR construct that includes Bcl‐XL cDNA [70]. Testing this modified CAR‐T‐cell therapy in mice with multiple myeloma showed that the overexpression of Bcl‐XL led to more sustained disease control. Overexpressing Bcl‐XL and Bcl‐2 in CAR‐T cells boosts their expansion and effectiveness against tumors while decreasing depletion and apoptosis initiation. The researchers also discovered that CAR‐T cells with elevated Bcl‐XL levels, when combined with the venetoclax, exhibited a more pronounced therapeutic effect compared to CAR‐T cells overexpressing Bcl‐2 [49]. This discovery points to a potential combination therapy approach, focused on improving CAR‐T cell persistence and inducing tumor cell apoptosis.

#### 4.1.2 Targeting Caspases to Inhibit Apoptosis

Caspases are essential regulators of apoptosis. Researchers observed that inhibiting caspase‐3 expression through electrotransfer‐mediated TCR knockdown in T cells significantly enhanced CAR‐T cell viability without compromising their function [71]. Consequently, lowering the transcription and activity of critical caspases represents a viable approach to improve CAR‐T cell survival. Utilizing CRISPR/Cas9 technology to efficiently knock out key caspases, like caspase‐3, during the creation of CAR‐T cells, the therapeutic impact of CAR‐T‐cell therapy could be significantly enhanced.

#### 4.1.3 Targeting the JAK–STAT Pathway to Inhibit Apoptosis

The JAK–STAT pathway is a well‐preserved signaling mechanism activated by cytokines, which are signaling molecules that help cells communicate. This pathway plays a vital role in regulating many essential cellular functions. It helps control how cells grow, move, and develop, as well as how they undergo programmed cell death, known as apoptosis. By transmitting signals from the cell surface to the nucleus, the JAK–STAT pathway ensures that cells respond appropriately to their environment and maintain normal function. Notably, JAK–STAT signaling is also crucial in modulating immune responses, highlighting its importance in CAR‐T‐cell therapy [72]. The connection between the JAK–STAT signaling pathway and apoptosis is intricate and context‐dependent, varying according to the specific cell type and the cytokines or growth factors involved. IFN‐I not only triggers apoptosis in tumor cells by activating JAK–STAT1 but also induces apoptosis in CAR‐T cells. To avoid the restriction of IFN‐I on CAR‐T cells, combining IFN‐I oncolytic viruses with interferon‐insensitive CAR‐T cells is essential [73]. Conversely, the JAK–STAT3/5 pathway enhances Bcl‐2 and Mcl‐1 expression, thereby effectively inhibiting apoptosis. In laboratory experiments, CAR‐T cells engineered with the 28‐ΔIL2RB‐z(YXXQ) construct continuously proliferate and cease terminal differentiation by enhancing the activity of the JAK‐STAT3/5 signaling pathway [74]. When compared to CAR‐T cells containing only the 4‐1BB or CD28 costimulatory domains, the YXXQ CAR‐T cells exhibited stronger antitumor effects and maintained their persistence longer in both liquid and solid tumor models. CAR‐T cells engineered with the BB‐trIL2RB‐z(YRHQ) motif effectively suppressed the progression of breast cancer and ovarian cancer at low doses through the JAK‐STAT3/5 pathway. Moreover, YRHQ CAR‐T‐cell therapy did not elicit grievous organ toxicity or inflammatory responses in vivo [75].

### 4.2 Strategies Targeting the Extrinsic Pathway to Inhibit Apoptosis

#### 4.2.1 Targeting FAS to Inhibit Apoptosis

In addition to activating apoptosis‐related pathways in tumor cells, it can also enhance the survival of CAR‐T cells, thereby improving their resistance to therapeutic challenges. To enhance the cytotoxicity of CAR‐T cells via apoptotic pathways (Figure 2), Ranjan and colleagues found that the death receptor Fas and the downstream molecule FADD are essential for regulating CAR‐T‐cell function [46]. Additionally, researchers engineered Fas decoys to counteract the suppression of CAR‐T cells caused by FasL, thereby increasing cytokine secretion and improving tumor cell elimination. Moreover, the introduction of Fas decoys significantly increased the durability and effectiveness of CAR‐T cells in xenograft models of pancreatic cancer [76]. Moreover, CAR‐T cell‐secreted IFN‐I can enhance surface Fas expression of tumor cells [46]. Evgin and colleagues found that infection of murine EGFRvIII CAR‐T cells with SVmIFNβ can enhance IFN‐I expression, which increases the levels of Fas and caspases via JAK‐STAT1 pathway to induce tumor cell death [73]. In addition, it is unknown whether any latent or delayed adverse events are relevant to the prolonged use of oncolytic viruses in CAR‐T‐cell therapy. Therefore, it is essential to conduct ongoing and meticulous monitoring over extended periods.

#### 4.2.2 Targeting TNFR1 to Inhibit Apoptosis

The effectiveness and longevity of CAR‐T cells are notably influenced by the types of costimulatory domains incorporated into their design. Research has shown that CAR‐T cells with the 4‐1BB domain exhibit improved longevity relative to those utilizing the CD28 costimulatory domain [77, 78]. The study investigated how the 4‐1BB domain improves CAR‐T‐cell performance. When CAR‐T cells were grown alongside Nalm6 leukemic cells, the 4‐1BB signaling pathway played a crucial role in enhancing the survival of these CAR‐T cells. This pathway worked by boosting the recruitment of TRAF proteins, which then activated the NF‐κB signaling pathway. As a result of this activation, levels of pro‐apoptotic proteins such as Bim and caspase‐3 were lowered, contributing to increased CAR‐T cell longevity. Correspondingly, there was an upregulation of Bcl‐XL and Bcl‐2, consistent with decreased apoptosis [79, 80]. As a result, CAR‐T cells exhibited improved persistence and cytotoxicity, leading to extended overall survival in Raji‐bearing mice [81]. However, the 4‐1BB intracellular domain is associated with slower kinetics. Recent studies have identified that the CD79A/CD40 costimulatory structure activates the NF‐κB pathway while inhibiting apoptosis gene expression. CAR‐T cells incorporating CD79A/CD40 and targeting CD19 demonstrated superior cytotoxicity and proliferation in murine models of both B‐NHL and B‐ALL, outperforming those with CD28 or 4‐1BB domains [82, 83].

## 5 Overcome the Limitations of Tumor Microenvironment on Car‐T Cells

### 5.1 Removing the Limitations of MDSCs on CAR‐T Cells

Currently, the TME affects both the progression and persistence of cancers, particularly those of solid organs. Within this environment, myeloid‐derived suppressor cells (MDSCs) form a varied group of immune cells that have a major impact on suppressing immune responses. Researchers have demonstrated that MDSCs are associated with tumor progression by diminishing the activity of CAR‐T cells in experimental tumor models (Figure 3). Prajna et al. [84] found the JAK2‐STAT3 pathway to be a driver of MDSC proliferation in the liver, thereby undermining antitumor immunotherapy. This hypothesis was further confirmed in a murine liver metastasis model. The authors revealed that inhibiting STAT3 can trigger apoptosis in immunosuppressive MDSCs by reducing Bcl‐2 levels and activating Fas and BAX, thus enhancing CAR‐T cell antitumor activity. As previously discussed, the JAK/STAT3 axis supports the survival and proliferation of CAR‐T cells. However, STAT3 inhibition may compromise CAR‐T cell function. Such inhibition can activate the JNK, MAPK, and PI3K/Akt pathways, which may result in either pro‐apoptotic or anti‐apoptotic signaling, contingent upon the cellular environment [85]. The inhibition of STAT3 may activate the JNK pathway in CAR‐T cells without compromising their overall function. In addition, we constructed a CAR cassette with special motifs that can trigger the JNK‐STAT3/5 axis to promote CAR‐T cell survival. Nalawade et al. created MUC1/TR2.41BB CAR‐T cells with dual targeting capabilities [86]. These engineered cells are designed to recognize and attack breast cancer cells that express MUC1, as well as myeloid‐derived suppressor cells (MDSCs) that present TRAIL‐2. The aim is to address the negative effects of MDSCs on CAR‐T cell performance. Researchers confirmed that the TRAILR2.41BB domain incorporated into CARs fostered CAR‐T cell survival and limited tumor growth. The activated TRAIL pathway induces the apoptosis of numerous MDSCs. Although the expression of TRAILR2 was elevated, CD4+ and CD8+ T cells did not undergo enhanced apoptosis. This effect is attributed to elevated intracellular levels of c‐FLIP protein. Although c‐FLIP shares structural similarities with caspase‐8, it is deficient in the essential protease activity required to trigger apoptosis [86]. Tan et al. incorporated c‐FLIP into a Her‐2‐targeted CAR construct to shield CAR‐T cells from Fas‐mediated activation‐induced cell death (AICD). However, cytotoxicity was limited in mice with breast cancer when CAR‐T cells overexpressed c‐FLIP. Therefore, overexpressing c‐FLIP in CAR‐T cells may not be ideal for alleviating FasL‐mediated AICD [87].

### 5.2 Improving the Infiltration Ability of CAR‐T Cells Through the cGAS‐STING Pathway

The TME presents several significant challenges, such as abnormal vascularization and the downregulation of cell adhesion molecules. To mitigate these issues, the integration of targeted therapies with CAR‐T cell treatment can improve CAR‐T cell efficacy. This combination of drugs enhances the expression of chemokines and facilitates more effective infiltration of CAR‐T cells into tumor sites. Recent studies suggest that the cGAS‐STING pathway might have significant implications for CAR‐T cell therapy. By influencing the TME and modulating immune responses, this pathway could potentially enhance CAR‐T cell efficacy and overcome some of the current limitations in treatment. Understanding the dynamics of this pathway could open up new opportunities for enhancing CAR‐T cell therapies. Tumor cells frequently accumulate various genetic mutations, which may be caused by endogenous factors such as oxidative stress and metabolic byproducts or by exogenous factors like ultraviolet radiation, ionizing radiation, and chemical carcinogens. Under conditions of cellular damage or stress, mtDNA from mitochondria may be released into the cytoplasm, where it can work together with nuclear dsDNA to activate the cGAS‐STING pathway. When cGAS binds to dsDNA, it forms dimers that activate the enzyme, leading to the production of cGAMP. The cGAMP molecule interacts with STING located on the ER membrane, causing significant conformational changes that activate STING. Following its initial activation, STING moves to the Golgi apparatus. In this location, STING engages with TBK1 and IRF3. When IRF3 is activated, it triggers the production of interferons and various chemokines [88]. Type I and III interferons are vital in mobilizing the immune system to combat tumors by activating key immune cells, including NK cells, CTLs, and DCs. Once activated, NK cells and CTLs directly target and destroy tumor cells, while DCs enhance the immune response by efficiently presenting tumor antigens. Moreover, IFN‐I can trigger apoptosis in tumor cells by activating pro‐apoptotic proteins, contributing to the elimination of tumor cells. Combining anti‐hCD20 CAR‐T cells with a STING agonist C3 for treating xenograft solid tumor models results in superior antitumor immune effects compared to the effects of administering CAR‐T‐cell therapy in isolation [89]. In renal carcinoma models, blocking PARP, a crucial protein involved in repairing DNA damage, using the PARP inhibitor OLA leads to a buildup of cytosolic DNA. This accumulation triggers the cGAS‐STING signaling pathway in cancer cells. This pathway activation increases the levels of IFN‐I and chemokines CXCL10 and CCL5 (Figure 2), which attract CD8+ CAR‐T cells to the tumor location. The CAR‐T cells then accumulate in the TME and release granzyme B, a potent cytotoxic molecule that induces apoptosis in cancer cells. Thus, PARP inhibition triggers a robust immune response in renal carcinoma models, significantly enhancing CAR‐T cell antitumor immunity and offering a promising new strategy for overcoming therapeutic resistance [90]. A low dose of the FEN1 inhibitor SC13 increases cytoplasmic dsDNA levels in cancer cells. This increase of dsDNA triggers the cGAS‐STING signaling pathway, which in turn boosts the production of chemokines. Enhanced chemokine production improves the infiltration of CAR‐T cells into solid tumors. Consequently, SC13 and other FEN1 inhibitors can enhance CAR‐T‐cell therapy by overcoming the issue of inadequate CAR‐T cell infiltration in solid tumors [91]. Nevertheless, further extensive clinical trials are essential to determine if activating the cGAS‐STING pathway can enhance the effectiveness of CAR‐T cells in treating tumors in patients.

### 5.3 Overcoming the Limitation of the cGAS‐STING Pathway on CAR‐T Cells

While the cGAS‐STING pathway shows potential for improving CAR‐T cell infiltration into solid tumors and enhancing therapeutic outcomes through mechanisms such as increased chemokine production and IFN‐I‐mediated immune activation, it is essential to address the associated limitations. Specifically, the activation of the cGAS‐STING pathway can lead to the production of IFN‐I and other factors that may adversely affect CAR‐T cell survival and functionality. Therefore, developing strategies to mitigate the negative impact of IFN‐I on CAR‐T cells is crucial. Addressing these challenges is key to optimizing CAR‐T cell therapy and achieving more effective and durable anti‐tumor responses.

The endogenous cGAS‐STING‐driven IFN‐I/III signaling is essential for achieving persistent and strong antitumor responses during CAR‐T cell immunotherapy. IFN‐I plays a crucial role in eliminating tumor cells by boosting the immune response against them [92, 93]. While IFN‐I fosters the immune system to attack tumors, it can simultaneously trigger the death of CAR‐T cells, potentially reducing their effectiveness in ongoing therapy. Therefore, strategies aimed at minimizing the harmful effects of IFN‐I on CAR‐T cells are essential to enhance the overall success of CAR‐T‐cell therapy. Researchers have found that the cGAS‐STING pathway also initiates an alternative form of NF‐κB signaling. Researchers have discovered that activating the cGAS‐STING pathway also triggers an alternative form of NF‐κB signaling [91, 92]. This process involves the movement of RELB, a specific subunit of NF‐κB, into the cell nucleus, where it plays a role in the signaling cascade. They have consequently identified a transcriptional regulator involving RELA that not only triggers the NF‐κB pathway but also significantly enhances the production of interferons IFN‐I and IFN‐III in CAR‐T cells (Figure 3). The NF‐κB pathway activates the expression of genes that inhibit apoptosis, such as Bcl‐XL and Bcl‐2. These proteins play a protective role in CAR‐T cells by counteracting the cell death signals induced by IFN‐I. Interestingly, CAR‐T cells co‐expressing the RELA K5R variant demonstrated a much higher efficacy in tumor cell eradication compared to CAR‐T cells without this variant [94]. Additionally, IFN‐I has the capacity to initiate the JAK‐STAT pathway.

## 6 Clinical Trials With Strategies for Apoptosis

For the past few years, a series of novel methods that target apoptotic pathways have entered various stages of clinical trials (Table 1). A currently ongoing phase 1/2 clinical trial (NCT05370547) is assessing the potential benefits of combining chidamide with CAR‐T‐cell therapy in treating 120 patients with relapsed or refractory (R/R) B‐NHL. Chidamide, a type of HDAC inhibitor, is being tested for its ability to enhance the effectiveness of CAR‐T‐cell treatment. In animal experiments, the investigators reported that CAR‐T‐cell resistance is linked to low Noxa expression and found that HDACi can upregulate Noxa expression in tumor cells. NOXA can downregulate MCL‐1 levels to increase the activity of BAX and BAK, which can promote apoptosis in tumor cells. Therefore, this trial will further investigate the impact of chidamide intervention on Noxa expression in patients. A phase 1/2 experiment (NCT04553393) will be conducted in aggressive R/R NHL patients with a large tumor burden, where they will be treated with decitabine‐primed tandem targeting CD19 and CD20 CAR‐T cells along with epigenetic agents. This trial demonstrated that in combination with HDACi, these compounds can induce tumor cell apoptosis, upregulate immune surveillance, and enhance CAR‐T‐cell therapy efficacy. The clinical trial NCT06481241 is designed to evaluate the efficacy and safety of combining venetoclax, a BCL‐2 inhibitor, with CAR‐T‐cell therapy for patients newly diagnosed with Philadelphia Chromosome‐negative B‐cell Acute Lymphoblastic Leukemia (Ph‐B‐ALL). Venetoclax is integral to this approach as it promotes apoptosis in tumor cells by inhibiting BCL‐2, which normally prevents cell death. By incorporating venetoclax into the treatment plan, the trial aims to boost the apoptotic response and enhance the CAR‐T cells' effectiveness in eliminating leukemia cells. This combination could potentially reduce the need for extensive chemotherapy, lowering treatment‐related toxicity and improving patient outcomes. In preclinical studies, researchers have shown that a new pan‐T booster, which combines a CD40 agonist with a T cell costimulator, enhances the antitumor activity of MSLN CAR T cells compared to previously reported MSLN CAR T cells. This innovation leverages CD40 co‐stimulation to activate the NF‐κB pathway, boosting CAR‐T cell proliferation, reducing cell death, and improving therapy effectiveness. In the clinical trial NCT05693844, patients receive an initial dose of these advanced pan‐T booster MSLN CAR T cells to assess their safety, feasibility, pharmacokinetics, pharmacodynamics, and overall efficacy in treating cancer.

**TABLE 1:** Clinical trials of methods targeting apoptotic pathways to improve CAR‐T cell therapy.

Disease	Intervention/treatment	Apoptotic pathways targeted	Status	Identifier	Trail phase	*n*
Non‐Hodgkin's Lymphoma	Drug: Chidamide Drug: Fludarabine and cyclophosphamide Biological: Anti‐CD19 CAR‐T cells	NOXA	Recruiting	NCT05370547	Phase1 Phase2	120
Refractory or Relapsed Aggressive r/r B‐NHL With Huge Tumor Burden	Drug: Chidamide Drug: Decitabine Drug: Chidamide and Decitabine Biological: Decitabine‐primed Tandem CAR19/20 engineered T cells	NOXA	Unknown status	NCT04553393	Phase1 Phase2	80
Newly Diagnosed Adult Patients with Ph‐B‐ALL	Combination product CAR‐T cells Drug: Venetoclax	Bcl‐2	Not yet recruiting	NCT06481241	Phase3	77
Advanced/Metastatic Solid Tumors	Biological: Pan‐T booster co‐expressing MSLN CAR T cell Drug: Albumin‐bound paclitaxel Drug: Cyclophosphamide Drug: Fludarabine	NF‐κB pathway	Recruiting	NCT05693844	Phase1 Phase2	15

## 7 Conclusion

Understanding the intricate mechanisms of apoptotic pathways is essential for advancing CAR‐T‐cell therapy and overcoming cancer resistance. A strategic focus on sensitizing tumor cells to CAR‐T‐cell‐mediated apoptosis could substantially enhance therapeutic efficacy. This can be achieved through combination therapies that incorporate BH3 mimetics, Mcl‐1 inhibitors, IAP inhibitors, and HDAC inhibitors. These agents suppress anti‐apoptotic proteins and activate intrinsic mitochondrial pathways, thereby increasing tumor cells' susceptibility to apoptosis. Additionally, compounds such as SP2509, indomethacin, and COX‐2 inhibitors can upregulate death receptor expression on tumor cells, thereby further promoting apoptosis and enhancing CAR‐T‐cell efficacy. Another crucial approach involves enhancing the cytotoxicity of CAR‐T cells. Resistance often emerges due to the upregulation of death ligands and inhibitory receptors on CAR‐T cells, thereby compromising their therapeutic potential. To counteract this, genetic engineering can be utilized to introduce constructs that promote the expression of anti‐apoptotic proteins such as Bcl‐2 and Bcl‐XL, as demonstrated in preclinical cancer models. These proteins further mitigate the adverse effects of venetoclax on CAR‐T cells, thereby extending their survival. Moreover, CAR‐T cells engineered with the YXXQ/YRHQ motif can activate the JAK‐STAT3/5 pathway, thereby maintaining their antitumor efficacy. Additionally, therapeutic strategies such as Fas decoys can downregulate death receptor expression on tumor cells, thereby preventing CAR‐T‐cell apoptosis. Our findings suggest that enhancing tumor cell sensitivity to apoptosis, in conjunction with augmenting CAR‐T cell cytotoxicity through targeted manipulation of apoptotic pathways, may lead to a significant improvement in therapeutic outcomes.

The TME presents significant challenge to CAR‐T‐cell therapy. MDSCs within the TME impair CAR‐T‐cell function. Dual‐targeting strategies, such as MUC1/TR2.41BB CAR‐T cells, which target both tumor‐specific antigens and MDSCs, show promise in maintaining CAR‐T‐cell efficacy even when amidst antigen mutations or reductions. Additionally, the limited trafficking and infiltration of CAR‐T cells into solid tumors are significant barriers. Agents like PPAR inhibitors, SC13, and FEN1 inhibitors can stimulate the production of cytokines like CXCL10 and CCL5, which recruit CAR‐T cells to the tumor site and enhance their infiltration. PPAR inhibitors can also activate the cGAS‐STING pathway in tumor cells, leading to increased IFN production and mobilization of surrounding immune cells. These strategies aim to neutralize the immunosuppressive characteristics of the TME, thereby improving CAR‐T‐cell efficacy.

While targeting apoptotic pathways offers promising strategies for overcoming CAR‐T‐cell resistance, further research is needed to evaluate the effectiveness of combining gene‐edited CAR‐T cells with conventional therapies. Despite their potential, challenges such as clinical feasibility and safety of these combinations remain. A deeper understanding of apoptotic pathways could improve CAR‐T cell persistence in patients and address intrinsic resistance mechanisms within cancer cells. By continuously innovating and refining these strategies, we have the potential to overcome resistance, significantly enhance CAR‐T‐cell therapy outcomes, and offer renewed hope to cancer patients globally.

## Author Contributions

**Zhanna Zhang:** conceptualization (lead), writing – original draft (lead), writing – review and editing (lead). **Manqi Su:** writing – original draft (supporting). **Panruo Jiang:** writing – original draft (supporting). **Xiaoxia Wang:** writing – original draft (supporting). **Xiangmin Tong:** writing – review and editing (supporting). **Gongqiang Wu:** conceptualization (supporting), writing – review and editing (supporting).

## Conflicts of Interest

The authors declare no conflicts of interest.

## Data Availability

Data sharing not applicable to this article as no datasets were generated or analysed during the current study.
